# Mandibular Ameloblastoma in an Elderly Patient: A Case Report

**DOI:** 10.1155/2013/145282

**Published:** 2013-03-06

**Authors:** Kokoro Nagata, Kasumi Shimizu, Chu Sato, Hiroshi Morita, Yoshihiro Watanabe, Toshiro Tagawa

**Affiliations:** Departments of Oral and Maxillofacial Surgery, and Clinical Sciences, Medical Life Science Mie University Graduate School of Medicine, 2-174 Edobashi, Tsu, Mie 514-8507, Japan

## Abstract

Ameloblastomas frequently occur in relatively young people, but are rarely seen in people aged 80 years or older. We report a case of mandibular ameloblastoma in an elderly patient with a review of the literature. The patient was a 82-year-old man who noticed swelling of the gingiva approximately 2 weeks prior to his initial visit. Computed tomography showed a radiolucent area with little radiopacity. Internal uniformity was observed at the site, with thinning of cortical bone which lacked continuity in some areas. The excision and curettage were performed under general anaesthesia. No recurrence has been observed 14 months after surgery.

## 1. Introduction

Among odontogenic tumours, ameloblastomas have the highest rate of occurrence after odontomas [1]. They are said to comprise between 10% and 50% of all odontogenic tumours [2–4]. The age group predilection peaks in the 20s and 30s, with the average age being between 30 and 40 years, and the majority of cases occur in the 30 to 60 years age group [1, 2, 5–7]. Based on these figures, ameloblastomas are considered to be fairly rare in the elderly. We present a case of ameloblastoma in the mandible of an 82-year-old man and discuss the occurrence of this tumour in the elderly. 

## 2. Case Presentation

An 82-year-old Japanese man presented with swelling of the gingival in the molar region of the left mandible. Approximately 2 weeks prior to the first visit, the patient noticed swelling of the gingiva, and panoramic X-rays were taken at a dental clinic. The images revealed radiolucent findings at the site, and the patient was referred to our facility for examination. The patient had a moderate physique and was well nourished, but he was taking medication for hypertension. 

**Figure 1:**
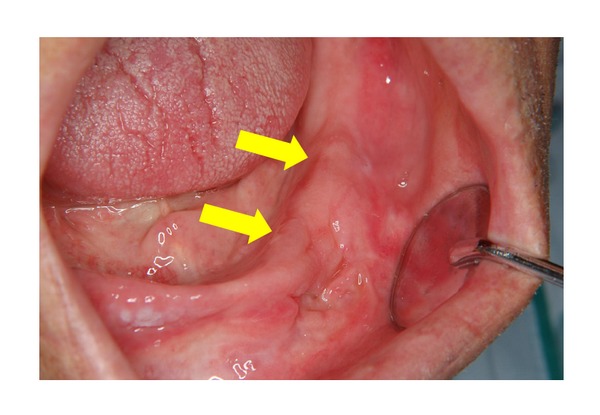
Intraoral findings at the first visit. The upper and lower jaws were edentulous, with a relatively irregular border from the centre of the mandible to the gingiva of the molar region on the left side. Diffuse swelling was observed, accompanied by surface ulceration.

Intraoral findings showed that the upper and lower jaws were edentulous, with a relatively irregular border from the centre of the mandible to the gingiva of the molar region on the left side. Diffuse swelling and surface ulceration were observed. There was neither tenderness nor numbness of the lips (Figure 1).

**Figure 2:**
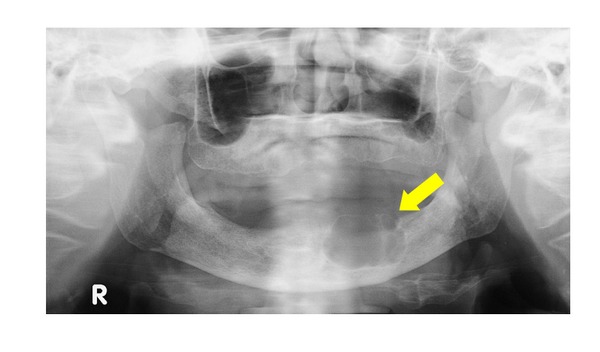
Panoramic X-ray view. A polycystic radiolucent area with an irregular margin can be seen in the molar region of the mandible on the left side.

Panoramic radiographs revealed a barely perceptible, polycystic radiolucent area with slightly irregular margins in the left molar region of the mandible (Figure 2).

**Figure 3:**
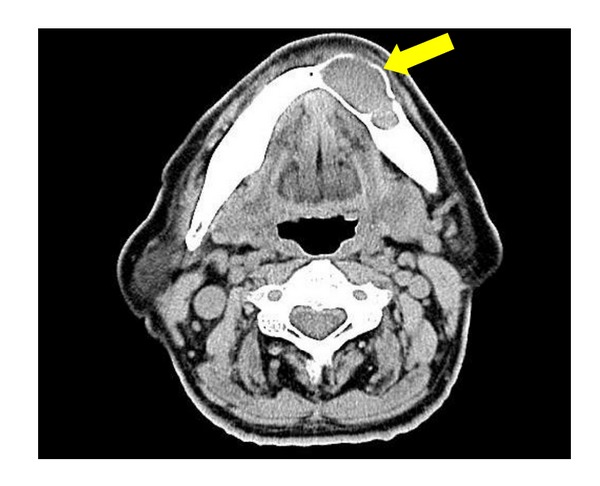
Computed tomography view. This shows thinning of peripheral bone was observed, with the bone lacking continuity in some areas.

Computed tomography showed a radiolucent area with little radiopacity. Internal uniformity was observed at the site, with thinning of cortical bone which lacked continuity in some areas (Figure 3). After 1 month, a biopsy and a needle aspiration were performed. Five millilitres of yellow-white content was aspirated. The results of bacteriological analysis were negative. Histopathological findings revealed that the squamous epithelium was accompanied by chronic inflammatory cell infiltration. Based on these findings, a diagnosis of benign tumor of the mandible was made, and after 2 months, excision and curettage were performed under general anaesthesia. The lesion partially adhered to the bone, and the surface of the peripheral bone was slightly rough. An inferior alveolar neurovascular bundle was also observed below the tumour, and this was preserved. After excision of the tumor in one piece, curettage was performed and the wound was left open. Seven months after surgery, there has been no recurrence of the tumour, and the patient is currently being monitored as an outpatient.

**Figure 4:**
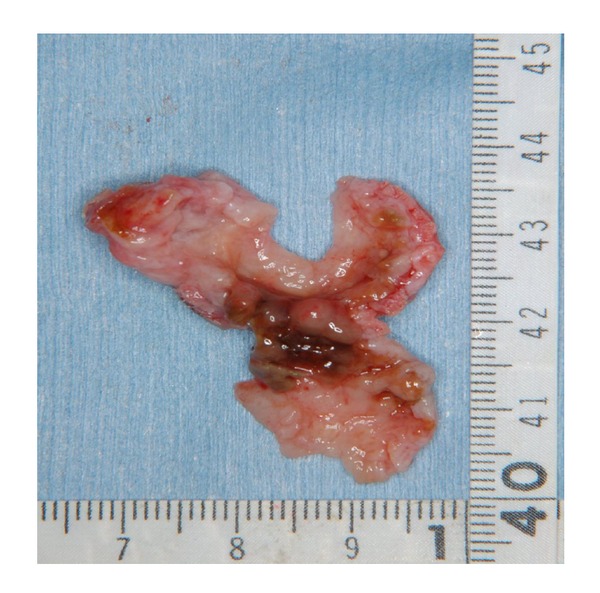
The excised tumor. It was milky-white in colour, with a slightly rough surface. The transverse section was largely cystoid, but solid portions were also observed.

**Figure 5:**
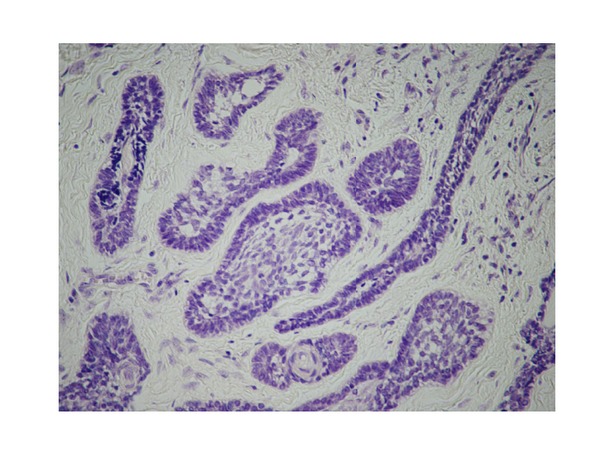
Histopathological findings. Alveolar cell hyperplasia with a funicular structure in the fibrosing interstitial tissue was observed, as well as a palisade arrangement (Haematoxylin and eosin staining, ×150).

The extracted tumour measured 3 × 2.5 cm and was milky-white in colour, with a slightly rough surface. The transverse section was mostly cystoid, but solid portions were also observed (Figure 4). Histopathological diagnosis was follicular-type ameloblastoma. Haematoxylin-eosin staining revealed alveolar cell hyperplasia with a funicular structure in the fibrosing interstitial tissue, as well as a palisade arrangement (Figure 5).

## 3. Discussion

Ameloblastomas have a relatively high rate of occurrence and are seen across a wide spectrum of ages [1]. The peak occurrence rate is in the 20s and 30s and reports of these tumours in elderly patients are rare [2, 5–7]. A thorough search by the authors found only 11 reports cases of ameloblastoma worldwide in patients aged 80 or older between 1977 and 2010. In six of these cases, including the case reported here, the ameloblastoma was located in the centre of the jaw [8–12], and there were three cases each in the maxilla and the mandible. In terms of histological type, three cases were of the follicular type, two were desmoplastic, and one was plexiform (Table 1). The five remaining cases were peripheral ameloblastomas [13–17], two of which occurred in the maxilla, one in the mandible or gingiva, and two in the buccal mucous (Table 2). The ratio of men to women in these 11 cases was 4 : 7, although this may be related to the longer life expectancy of women than men. Of the 11 cases, eight cases were Japanese patients, one was of Asian ethnicity, one was African, and in one case the ethnicity was not noted. Reichart et al. categorised ameloblastoma patients into three ethnicities and found that the mean ages at the time of diagnosis were 28.7, 39.9, and 41.2 years for patients of African, Caucasian and Asian ethnicity, respectively [6]. The fact that the age at occurrence among Asian patients is higher may be because more than 80% of the cases in patients 80 years of age or older occur in Asians.

In reports by Philipsen et al. [18] and Wettan et al. [19], peripheral ameloblastoma comprises 1–10% of all ameloblastoma cases, but in our study of elderly patients, five of the 11 cases studied (45.5%) were peripheral ameloblastomas. According to a 2005 classification by WHO, the mean age of occurrence for intraosseous ameloblastoma is 37 years, while the mean age of patients with peripheral ameloblastomas is 51 years, and 64% of all cases occur between 50 and 70 years of age [1]. This advanced age may reflect the fact that the age of occurrence is higher for peripheral ameloblastomas. Based on these facts, intraosseous ameloblastomas in elderly patients, as in the case described here, are believed to be relatively rare. Moreover, it is possible that figures for peripheral ameloblastomas include those that occur as a result of alveolar bone being absorbed as a function of ageing, in which case the tumour remains in the soft tissue.

Among reported cases of intraosseous that were diagnosed preoperatively, there have been three cases, including the one described here, in which a benign tumour was suspected [8, 10]: one case of cyst [9], one case in which a clear diagnosis could not be made [11], and one case of malignant tumour [12]. When diagnosing ameloblastomas in elderly patients, there is frequently missing or defective dentition in the affected area, and there are no typical signs such as root absorption. Thus, it is important to differentiate patients with tumours from those with cystic diseases such as residual cysts.

A number of therapies are being investigated for the treatment of ameloblastomas, but in elderly patients, considering their overall physical condition and age, it is necessary to select a minimally invasive surgical approach. Even when the tumour was resected, resection was sometimes performed under local anaesthesia, due to the patient's overall physical condition and the need to preserve the function of the affected area [14] and cases in which the tumour was resected to the greatest possible extent [9]. In the case described here, after a diagnosis of ameloblastoma was confirmed by intraoperative frozen section diagnosis, the tumour was extracted and curettage was performed. Repeat curettage would generally be performed after 6 months or 1 year, but instead the patient was monitored, due to his advanced age. The authors believe that ongoing, close monitoring will be necessary for this patient.

## Figures and Tables

**Table 1:** Intraosseous ameloblastoma.

Year	Author	Sex	Age	Location	Pathological type	Treatment	Race
1986	Ohyama et al. [8]	M	82	Maxilla	Follicular type	Excision (general anesthesia)	Japanese
1991	Iwata et al. [9]	F	83	Maxilla	Follicular type	Excision (general anesthesia)	Japanese
1998	John et al. [10]	F	80	Maxilla	Plexiform type	Excision (general anesthesia)	Black
1998	Lee et al. [11]	F	83	Mandible	Desmoplastic type	Marginal resection	Asian
2004	Koya et al. [12]	M	81	Mandible	Desmoplastic type	Marginal resection (general anesthesia)	Japanese
2010	This case	M	82	Mandible	Follicular type	Excision and curettage (general anesthesia)	Japanese

**Table 2:** Peripheral ameloblastoma.

Year	Author	Sex	Age	Location	Treatment	Race
1977	Frankel et al. [13]	F	92	Maxillary gingiva	Excision (general anesthesia)	Not noted
1988	Takeda et al. [14]	F	89	Mandibular gingiva	Excision and curettage (local anesthesia)	Japanese
1992	Ohuchida et al. [15]	F	81	Maxillary gingiva	Excision (general anesthesia)	Japanese
2007	Yamanishi et al. [16]	M	80	Buccal mucosa	Excision (general anesthesia)	Japanese
2009	Isomura et al. [17]	F	88	Buccal mucosa	Excision (general anesthesia)	Japanese

## References

[1] Gardner DG, Heikinheimo K, Shear M, Philipsen HP, Coleman H, Barnes L, Eveson JW, Reichart P, Sidransky D (2005). Ameloblastomas. *World Health Organization Classification of Tumors. Pathology and Genetics of Head and Neck Tumors*.

[2] Takagi M (2004). *Atlas of Oral Pathology*.

[3] Daley TD, Wysocki GP, Pringle GA (1994). Relative incidence of odontogenic tumors and oral and jaw cysts in a Canadian population. *Oral Surgery, Oral Medicine, Oral Pathology*.

[4] Kasahara K, Kobayashi I, Fujiwara T (1994). Clinical Study of the odontogenic tumors. *Journal of the Japan Stomatological Society*.

[5] Robert EM, Diane S (2003). *Oral and Maxillofacial Pathology*.

[6] Reichart PA, Philipsen HP, Sonner S (1995). Ameloblastoma: Biological profile of 3677 cases. *European Journal of Cancer B*.

[7] Gardner DG (1999). Critique of the 1995 review by Reichart et al. of the biologic profile of 3677 ameloblastomas. *Oral Oncology*.

[8] Ohyama S, Koga N, Koga M (1986). Ameloblastoma of the maxilla: report of a case. *Japanese Journal of Oral and Maxillofacial Surgery*.

[9] Iwata M, Nishijima K, Takagi S (1991). Ameloblastoma of the maxilla: report of four cases and review of the literature. *Japanese Journal of Oral and Maxillofacial Surgery*.

[10] John G, Stewart KL, Steven RI, Julius RB, Marshall PS (1998). Plexiform ameloblastoma involving the maxillary antrum. *The New York State Dental Journal*.

[11] Lee CY, Lee J, Hirata K, Tomich CE (1998). Desmoplastic variant of ameloblastoma in an 83-year-ald Asian female: report of a case with literature review. *Hawaii Dental Journal*.

[12] Koya E, Mihara M, Nakashiro K, Shintani S, Hamakawa H (2004). A case of desmoplastic ameloblastoma in an elderly patient. *Japanese Journal of Oral Diagnosis and Oral Medicine*.

[13] Frankel KA, Smith JD, Frankel LS (1977). Soft tissue ameloblastom in a 92-year-old woman. *Archives of Otolaryngology*.

[14] Takeda Y, Kuroda M, Suzuki A (1988). Ameloblastoma of mucosal origin. *Acta Pathologica Japonica*.

[15] Ohuchida M, Tanaka S, Kusukawa J, Nagata A, Okina T, Kameyama T (1992). A case of peripheral ameloblastoma arising in the maxilla. *Japanese Journal of Oral and Maxillofacial Surgery*.

[16] Yamanishi T, Ando S, Aikawa T (2007). A case of extragingival peripheral ameloblasotoma in the buccal mucosa. *Journal of Oral Pathology and Medicine*.

[17] Isomura ET, Ishimoto S, Yamada T, Ono Y, Kishino M (2009). Case report of extragingival peripheral ameloblastoma in buccal mucosa. *Oral Surgery, Oral Medicine, Oral Pathology, Oral Radiology and Endodontology*.

[18] Philipsen HP, Reichart PA, Nikai H, Takata T, Kudo Y (2001). Peripheral ameloblastoma: biological profile based on 160 cases from the literature. *Oral Oncology*.

[19] Wettan HL, Patella PA, Freedman PD (2001). Peripheral ameloblastoma: review of the literature and report of recurrence as severe dysplasia. *Journal of Oral and Maxillofacial Surgery*.

